# The effect of hydroalcoholic extract of *Cichorium intybus *leaf on aryl hydrocarbon receptor expression in the testis of Wistar rats exposed to cigarette smoke

**DOI:** 10.22038/AJP.2022.21307

**Published:** 2023

**Authors:** Maryam Hashemi, Mahnaz Azarnia, Zahra Hajebrahimi, Samad Nejad Ebrahimi

**Affiliations:** 1 *Department of Animal Biology, Faculty of Biological Sciences, Kharazmi University, Tehran, Iran*; 2 *A&S Research Institute, Ministry of Science Research and Technology, Tehran, Iran* *Department of Phytochemistry, Medicinal Plants and Drugs Research Institute, Shahid Beheshti University, G. C., Evin, Tehran, IranDepartment of Phytochemistry, Medicinal Plants and Drugs Research Institute, Shahid Beheshti University, G. C., Evin, Tehran, Iran Department of Phytochemistry, Medicinal Plants and Drugs Research Institute, Shahid Beheshti University, G. C., Evin, Tehran, Iran Department of Phytochemistry, Medicinal Plants and Drugs Research Institute, Shahid Beheshti University, G. C., Evin, Tehran, Iran*; 3 *Department of Phytochemistry, Medicinal Plants and Drugs Research Institute, Shahid Beheshti University, G. C., Evin, Tehran, Iran*

**Keywords:** Cichorium intybus, Spermatogenesis, Malondialdehyde, Caspase-3, Cigarette smoke

## Abstract

**Objective:**

Cigarette smoke (CS) contains compounds such as reactive oxygen species (ROS). Oxidative stress caused by excessive ROS eventually leads to germ cell apoptosis and male infertility. The leaves of *Cichorium intybus *(chicory) are rich in natural antioxidants, but their protective effects on the adverse effects of CS on testicular tissue have not been studied.

**Materials and Methods:**

24 Wistar rats were classified into four groups: control, extract: treatment with chicory extract (200 mg/kg body weight/day) for 13 weeks, smoke: exposed to CS for 13 weeks, and smoke + extract: exposed to CS and treated with the C.* intybus* extract. Histological and biochemical analyses and apoptosis assay were done, and *Ahr,* and *Cyp1a1 *expression was determined.

**Results:**

Treatment with *C. intybus* compensated for the reduction of Sertoli cells, spermatogonia, spermatocytes, and spermatids caused by CS. Chicory extract reduced free radicals and improved antioxidant status. The lowest and highest percentage of apoptotic cells was observed in the extract and smoke groups, respectively, while simultaneous treatment with *C. intybus* extract led to a significant reduction of apoptotic cells. The mean *Ahr *levels in the control, extract, smoke and smoke + extract groups were 1.00±0.57, 1.93±0.25, 5.98±0.42, and 0.62±0.22, respectively (p˂0.05). The mean levels of *Cyp1a1* expression in the control, extract, smoke and smoke + extract groups were 1.00±0.31, 2.28±0.65, 5.55±0.40, and 0.21±0.23 (p˂0.05).

**Conclusion:**

The *C. intybus* extract probably affected* Cyp1a1* expression by downregulation of *Ahr. *These led to a decrease in free radicals and apoptosis, and an improvement in antioxidant status.

## Introduction

Smoking is one of the most important health problems, especially in reproductive age women and men, due to the detrimental effects on male and female fertility. In men, smoking reduces sperm count, impairs sperm motility, and lowers testosterone levels (Chohan and Badawy, 2010 ▶; Sharma, 2017 ▶). Toxic compounds in cigarette smoke (CS) accumulate in seminal plasma, thus affecting sperm motility, viability, morphology, and fertility in humans (Esakky et al., 2015b ▶). In rodents, chronic exposure to CS can arrest spermatogenesis, and CS compounds are potent toxins for meiotic spermatocytes (Zhao et al., 2019 ▶). CS is a strong stimulant of apoptosis because it contains compounds such as reactive nitrogen and oxygen species (RNS and ROS, respectively). Excessive ROS, especially with an inadequate antioxidant response, can cause oxidative stress, eventually leading to cell damage and apoptosis (Iu et al., 2017 ▶).

The role of the antioxidant pathway in the developmental homeostasis of male germ cells is so sensitive that both high activity and failure dealing with oxidative stress can lead to adverse outcomes on spermatogenesis and fertility. Close crosstalk of aryl hydrocarbon receptor (Ahr) with nuclear factor erythroid 2–related factor 2 (nrf2) and Hypoxia-inducible factor 1-alpha (HIF-1α) could indicate the important role of *Ahr* in regulating oxidative stress signaling and development of male germ cells (Zhang et al., 2020 ▶). Lack of Ahr and/or its activation leads to apoptosis, inflammation and DNA damage due to oxidative stress in sperm (Esakky and Moley, 2016a ▶).

Oxidative stress caused by CS plays an important role in male infertility (Taha et al., 2012 ▶). Therefore, antioxidant compounds may be a good treatment option for male infertility due to smoking. Some plants are rich in antioxidants and are used to treat diseases in different parts of the world due to their health-boosting properties (Akinrinde AS et al., 2022 ▶; Dorostghoal et al., 2019 ▶).

*Cichorium intybus*or (chicory) is used as a medicinal plant in folk medicine from South Asia to North Africa. In Iran, chicory leaves and seeds are used to treat liver diseases and purify the blood. In liver damage due to oxidative stress, the protective effect of *C. intybus *extract (lower concentrations) may be due to its antioxidant effects, inhibition of free radical production by inhibition of cytochrome P450 activity, purging of free radicals, or a combination of these mechanisms (Jamshidzadeh et al., 2006 ▶). Chicory leaves are rich in natural antioxidants that can meet nutritional and medicinal needs (Dorostghoal et al., 2019 ▶). The protective effects of *C. intybus* on the adverse effects of CS on testicular tissue have not been studied.

Therefore, we evaluated the effects of hydroalcoholic extract of *C. intybus* leaf on spermatogenesis, oxidative stress, and apoptosis in testes of adult male Wistar rats exposed to CS.

## Materials and Methods

**Plant materials**

The aerial parts of *C. intybus* cultivated in Qazvin Iran in 2017 were dried in shadow. The plant materials were identified by Dr. Ali Sonboli and a voucher specimen (MPH-2823) was kept at the herbarium of Medicinal Plants and Drugs Research Institute, Shahid Beheshti University, Tehran, Iran.

**Preparation of hydroalcoholic extract of aerial parts of** ***C. intybus***

 The aerial parts of *C. intybus *(900 g) were powdered using a mill and successively extracted three times with 6 L ethanol: water (70:30) at room temperature using a percolator (Pandey and Tripathi, 2014 ▶) The extraction process took 72 hr. The obtained liquid extract was filtered through a filter paper. The extract was evaporated at 35°C until dry using a rotary evaporator (Buchi, Switzerland) under vacuum. The 200-g gummy dark extract obtained was kept in a refrigerator (4°C) for HPLC analyses. 

The extract was analyzed with waters liquid chromatography equipment consisting of a 2695 Separations Module (USA), an autosampler equipped with a 100 μl loop, and Photodiode Array Detectors (PDA) using an RP- C18 SunFire column (3.5 μm, 3.0×150 mm i.d., Waters) equipped with a guard column (3×20 mm i.d.). The solvent system used a mixture of acetonitrile +0.1% formic acid (A) and H_2_O+0.1% formic acid (B), the following gradient was applied: 2% (A) during 10 min; 2% to 20% (A) in 20 min; 20% to 100% (A) in 10 min; 100% (A) in 6 min (time for all process: 36 min); the flow rate was 0.4 ml/min and the injection volume was 20 µl. The HPLC-UV chromatogram was recorded at 330 nm.

**Animals**

We conducted this study with the approval of the ethics committee of Kharazmi University (IR.KHU.REC.1400.007) using male Wistar rats (n=6 / group, 270-320 g). Rats were kept in the animal room of the Faculty of Biological Sciences, Kharazmi University, Iran. The animals were kept in fiberglass cages at 22±2°C and 12 hr of darkness/12 hr of light with access to water and food.

**Experimental design**

**Figure 1:**
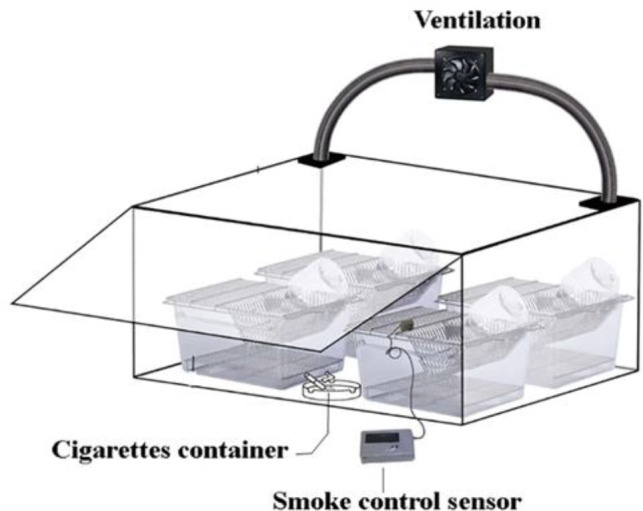
The smoke chamber

To create a model of rats exposed to CS, a smoke chamber was used (Figure 1) and the animals were restricted and exposed to CS by a smoking machine. A syringe sucks the smoke from lighted cigarettes and pumps it around the rat compartment. In the smoke machine, by imitating human smoking, each cigarette lasted 8-10 min (Khanna et al., 2013 ▶). The total period was thirteen weeks, during which, the animals were exposed to CS for six days per week. Thirty cigarettes were smoked alternatively three times a day. This process was performed for rats in the smoke and smoke + extract groups. Animals in the control and extract groups were placed in the chamber for the same period of time without exposure to smoke. The four experimental groups were: control (without any treatment), extract (treatment with chicory leaf extract 200 mg/kg body weight/day (Dorostghoal et al., 2019 ▶) for 13 weeks (Mahaneem et al., 2011 ▶), gavage), smoke (the treatment method as described above), and smoke + extract (exposed to CS (similar to the smoke group) and treated with the extract (as in the extract group, and before exposure to cigarette smoke)). Commercially available cigarettes (Zica, Iranian Tobacco Company, Iran) containing about 0.8 mg nicotine and 11 mg tar per cigarette, were used for all CS exposures. At the end of the treatment period, rats were anesthetized by intra-peritoneal injection of ketamine (80 mg/kg) and xylazine (10 mg/kg) (Alfasan, Woerden, and Holland) and testes were collected for histological examination, expression of caspase-3 protein, and *Ahr* and *Cyp1a1* genes expression.

**Histological analysis**

To prepare the tissue for histological analysis, the testis was fixed in Bouin's solution for 20 hr. After tissue processing, sections with a thickness of 5 μm were prepared. The slides were stained by hematoxylin-eosin and the number of cells was counted using a Graticule field of view with a 100x object lens. Twenty fields were counted for each animal. Round or near-round seminiferous ducts were randomly selected in each field and spermatogonia, spermatocytes, and spermatids were counted. We used ImageJ software (LOCI, University of Wisconsin) to count and analyze data.

**Quantitation of malondialdehyde (MDA) and glutathione (GSH) levels**

 The levels of MDA and GSH in the testis of the studied groups were measured using ZellBio GmbH kits (#ZB-MDA-96A, and #ZB-GSH-96A, Germany, respectively). The steps were performed based on the instructions of the manufacturer. For this purpose, testes frozen at -70°C, which were uniform in the homogenizer, were used.

**Immunoperoxidase staining of caspase-3 for the detection of apoptotic cells**

 After preparing 5 μm sections of tissue fixed in Bouin's solution and embedded in paraffin, xylene was used twice for 5 min for deparaffinization. Rehydration was performed by descending ethanol series (100% for 3 min; 95, 80, and 70% for 1 min each). The samples were washed with distilled water. The slides were placed in an antigen retrieval buffer and placed in the microwave (three times, each for 20 sec). After cooling to room temperature, the slices were washed twice with phosphate-buffered saline with 0.1% Tween (PBS-T; 5 min each time). In order to inactivate endogenous peroxidases, the sections were incubated in 10% H_2_O_2_ (10 min). After incubating the samples in the blocking buffer (room temperature, 30 min), a caspase-3 antibody (#ab4051, Abcam) was added to the sections for 1 hr at room temperature. The slides were washed with PBS-T and horseradish peroxidase (HRP)-conjugated secondary antibody (#31A1067, Novus) was added in the dark (room temperature, 1 hr). The sections were washed again with PBS-T. To complete the reaction, 3, 3′-diaminobenzidine (DAB) solution (#300155400, Pro Taqs) was added as HRP substrate. Washing with PBS-T was performed to remove excess chromogen. The samples were dehydrated with ethanol 35, 70, 90% (20 sec each), and 100% (2 min), respectively, and clarified with xylene. The sections were mounted on glass slides and observed using light microscopy (Zeiss, Germany). 

**Total RNA extraction and cDNA synthesis**

 The testes were collected and instantly immersed in liquid nitrogen to protect RNA by inactivating RNase. Samples were stored at -80°C. Total RNA was isolated by means of the RiboEx kit (GeneAll Biotechnology, Korea). The quality of extracted RNA was evaluated via a nanodrop Spectrophotometer (Thermo Fisher Scientific, US). Suitable specimens for later steps had ratios of 260 nm: 280 nm≥1.8. cDNA synthesis was performed by using a Parstous kit (Iran) according to the manufacturer's instructions.

**Quantitative reverse transcription PCR**

 Two-step RT-qPCR was used to quantitatively analyze the expression of desired genes. After cDNA synthesis, we prepared a reaction solution based on the following ingredients: primer mixture (2 µl), cDNA (2 µl), and Real Q Plus 2X Master Mix (21 µl) (Amplicon, Denmark). The reaction solution was exposed to 50^°^C for 5 min, 95°C for 5 min, and 60-62°C (40 cycles, 30 sec each). The sequences of forward and reverse primers (Yamashita et al. 2014) are listed in Table 1. The desired protocol was given to the thermal cycler and after making the settings, the program was executed. The temperature program of the thermal cycler was as follows: initial denaturation (95°C, 5 min), denaturation (95°C, 15 sec), annealing (55-65°C, 25 sec, 40 cycles), extension (72°C, 25 sec), and final extension (65°C, 5 sec). 

**Statistical analyses**

 The software of Statistical Package for the Social Sciences (SPSS) version 22.0 was used for data analyses. Data are presented as average±SEM. Differences between groups were compared by one-way ANOVA and Tukey's test. Statistically significant levels were considered at p values< 0.05. For RT-qPCR, the expression ratio of the desired gene to the reference gene was calculated from the following formula, which is based on Efficiency and difference in C_t_.

$\mathrm{Ratio}=\frac{{\left(\mathrm{E target}\right)}^{\mathrm{\Delta CTtarget}(\mathrm{Control}-\mathrm{Sample}}}{{\left(\mathrm{E ref}\right)}^{\mathrm{\Delta CTref}(\mathrm{Control}-\mathrm{Sample})}}$

**Table 1:** The sequences of PCR primers

Product length (bp)	Accession number	Primer sequence (5´ to 3´)	Gene
130	NM_017008	Forward: AGGTTGTCTCCTGTGACTTC Reverse: CTGTTGCTGTAGCCATATTC	Glyceraldehyde-3-phosphate dehydrogenase (*Gapdh*)
100	NM_013149.2	Forward: CAGGCGTTCCTAAGCAAGTTTC Reverse: GGAGGTGAGCAGCAGTCTGA	Aryl hydrocarbon receptor (*Ahr*)
127	NM_012540.2	Forward: GTGGCCTGTATTTTGCTTATG Reverse: AGCTCAGGTACGTTTTTCCTA	Cytochrome P450, family 1, subfamily a, polypeptide 1 (*Cyp1a1*)

## Results

**HPLC-UV analysis of** ***C. intybus*** **extract**

**Figure 2:**
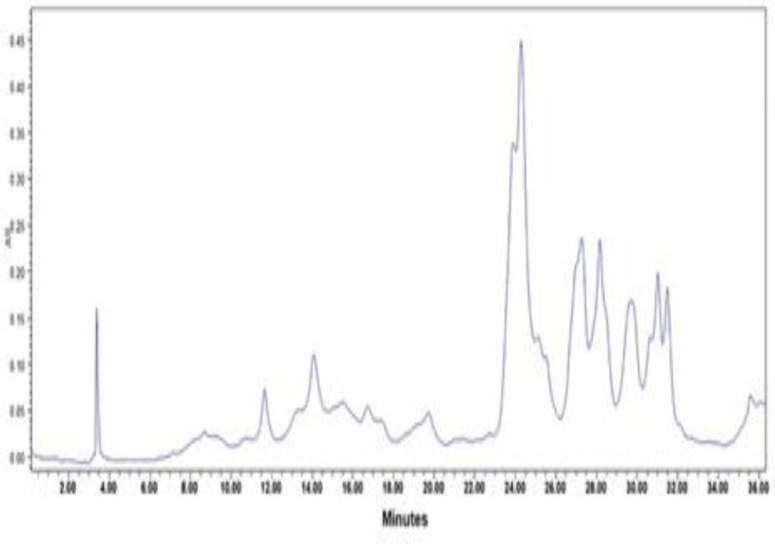
The HPLC-UV chromatogram of chicory hydroalcoholic extract at 330 nm

The HPLC-UV analysis of the hydroalcoholic extract of chicory is presented in Figure 2. The quantification of chicoric acid as a major compound revealed 468 µg/g in dry weight of plant material.

**Evaluation of the percentage of cells in testicular tissue**

After staining the testicular tissue with hematoxylin and eosin, Sertoli cells, spermatogonium, spermatocytes, and spermatids were counted in seminiferous tubules.

**Figure 3:**
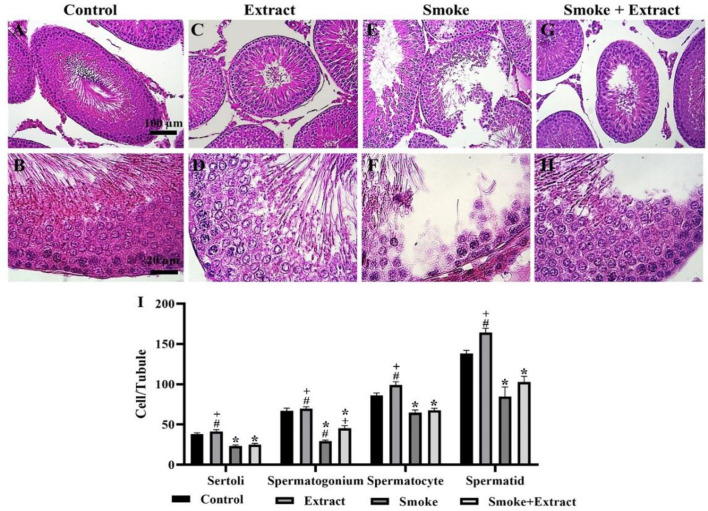
(A-H) Histological changes of testicular tissue after treatment with CS and *C. intybus* leaf extract. Exposure to CS led to a significant reduction of Sertoli cells, spermatogonia, spermatocytes, and spermatids. *C. intybus* leaf extract in combination with CS increased the mentioned cells. The increase in spermatogonia was significant compared to the CS group (scale bar of top row: 100 µm; scale bar of bottom row: 20 µm). (I) The histogram shows the number (mean±SEM) of mentioned cells per tubule in different groups (n=6 / group). Differences between groups were compared by one-way ANOVA and Tukey's test. CS: cigarette smoke. *statistically different from the control group (p<0.5), +statistically different from the smoke group (p<0.5), #statistically different from the smoke+extract group (p<0.5)

The number of Sertoli cells per seminiferous tubule in the control group, extract, smoke, and smoke + extract was 38.00±1.73, 41.33±2.33, 23.33±1.45, and 25.00±1.53, respectively. The smoke and smoke + extract groups were remarkably different from the control and extract groups (p˂0.05), while the two groups were not significantly different (Figure 3).

The number of spermatogonium cells per seminiferous tubule in the control group, extract, smoke, and smoke + extract was 67.00±3.22, 69.67±2.33, 29.33±1.45, and 45.33±3.18, respectively. The smoke and smoke + extract groups were considerably different from the control and extract groups (p˂0.05). The difference between the two groups of smoke and smoke + extract was also considerable (p˂0.05) (Figure 3).

The number of spermatocytes per seminiferous tubule in the control group, extract, smoke, and smoke + extract was 86.00±3.22, 99.00±4.16, 64.67±3.48, and 67.67±2.40, respectively. The smoke and smoke + extract groups were remarkably different from the control and extract groups (p˂0.05), while the two groups were not significantly different (Figure 3).

The number of spermatids per seminiferous tubule in the control group, extract, smoke, and smoke + extract was 138.00±4.16, 164.30±5.34, 84.67±11.84, and 102.70±7.26, respectively. The smoke and smoke + extract groups were significantly different from the control and extract groups (p˂0.05), while the two groups were not significantly different (Figure 3).

In all cases mentioned, the highest number of cells was observed in the seminiferous tubules of the extract group, on the other hand, the lowest number of cells per seminiferous tubule was observed in the smoke group. 

**Biochemistry of testicular tissue**

MDA level as a lipid peroxidation index showed a notable (p˂0.05) increase in the smoke group, while the difference in MDA levels between the other three groups was not significant (Table 2). Unlike MDA, CS significantly (p˂0.05) reduced GSH levels compared to the extract and smoke + extract groups. The highest level of GSH was observed in the extract group, which was significantly (p˂0.05) different from the other three groups (Table 2).

**Detection of apoptosis based on the presence of caspase-3 protein in testicular tissue**

**Figure 4:**
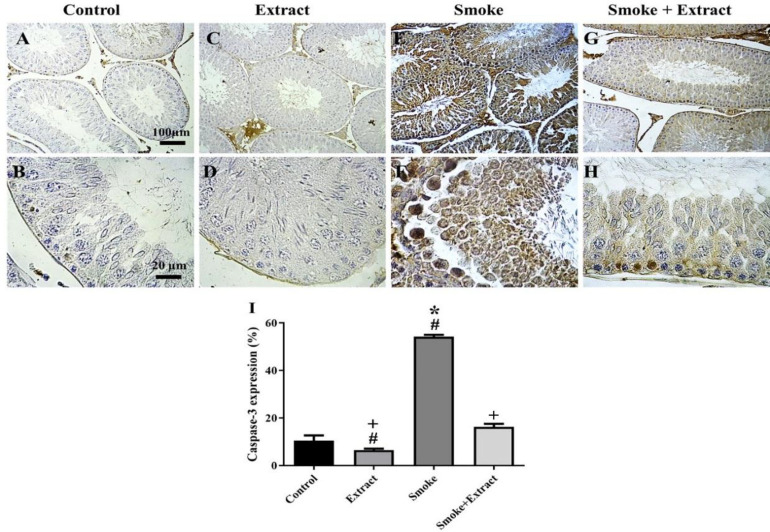
(A-H) Results of caspase-3 immunohistochemical staining after exposure to CS and treatment with *C. intybus* leaf extract. Exposure to CS significantly increased caspase-3 expression in testicular tissue of Wistar rats. *C. intybus* leaf extract in combination with CS significantly reduced caspase-3 expression. Caspase-3 expression was even lower in the extract group compared to the control group (scale bar of top row: 100 µm; scale bar of bottom row: 20 µm). (I) The histogram shows the percentage of caspase-3 expression (mean±SEM) in different groups (n=6 / group). Differences between groups were compared by one-way ANOVA and Tukey's test. CS: cigarette smoke. *statistically different from the control group (p<0.5), +statistically different from the smoke group (p<0.5), #statistically different from the smoke+extract group (p<0.5)

Caspase-3 is known as the primary executioner of programmed cell death or apoptosis. The highest percentage of caspase-3 expression was observed in the smoke group (54.25±0.79%), and was significantly different from the other three groups (p˂0.05). The lowest percentage of apoptotic cells was in the seminiferous tubules of the group that was treated only with chicory extract (6.48±0.64%). The expression level of caspase-3 in the extract group was remarkably different from the smoke and smoke + extract groups (p˂0.05). The percentage of caspase-3-expressing cells in the seminiferous tubules of the control group was 10.49±2.21% and was significantly different from the smoke group (p˂0.05). The percentage of apoptotic cells in the smoke + extract group was 16.34±1.23% and was considerably different from the extract and smoke groups (p˂0.05) (Figure 4).

**Table 2:** Effect of CS and *C. intybus* leaf extract on MDA and GSH levels in the testis of Wistar rats

**Smoke + Extract**	**Smoke**	**Extract**	**Control**	**Parameters**
0.81±0.08	1.80±0.26*	0.62±0.10	0.58±0.08	**MDA (**µmol/mg protein)
0.36±0.06+	0.15±0.02#	0.62±0.08*+#	0.29±0.04	**GSH** (mmol/mg protein)

Values are presented as mean±SEM (n=6/each group). Differences between groups were compared by one-way ANOVA and Tukey's test. *statistically different from the control group (p<0.5), +statistically different from the smoke group (p<0.5), #statistically different from the smoke+extract group (p<0.5). MDA: malondialdehyde, GSH: glutathione

**Expression levels of** ***Ahr*** **and** ***Cyp1a1*** **(Cytochrome P4501a1) in testicular tissue**

**Figure 5:**
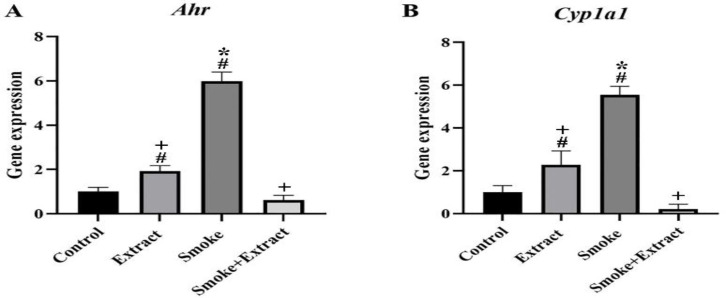
(A) *Ahr* and (B) *Cyp1a1* expression after exposure to CS and treatment with *C. intybus* leaf extract. CS and *C. intybus* leaf extract changed *Ahr* and *Cyp1a1* gene transcriptional activity in testicular tissue of Wistar rats. CS resulted in the upregulation of *Ahr* and *Cyp1a1 *in the smoke group. These modulations were significant in *Ahr *and *Cyp1a1* expression. Simultaneous treatment with *C. intybus* leaf extract resulted in a significant reduction in gene expression. Values are presented as mean±SEM (n=6 / group). Differences between groups were compared by one-way ANOVA and Tukey's test. *statistically different from the control group (p<0.5), +statistically different from the smoke group (p<0.5), #statistically different from the smoke+extract group (p<0.5). CS: cigarette smoke, *Ahr*: Aryl hydrocarbon receptor, *Cyp1a1*: Cytochrome P450, family 1, subfamily a, polypeptide 1

The expression levels of *Ahr *and *Cyp1a1 *changed after exposure to smoke and extract treatment compared with the control group. The mean *Ahr *levels in the control, extract, smoke and smoke + extract groups were 1.00±0.57, 1.93±0.25, 5.98±0.42 and 0.62±0.22, respectively. The difference between the extract, smoke, and smoke + extract groups was significant, but only the smoke group was considerably different from the control group (p˂0.05) (Figure 5 A).

The mean levels of *Cyp1a1* expression in the control, extract, smoke and smoke + extract groups were 1.00±0.31, 2.28±0.65, 5.55±0.40 and 0.21±0.23, respectively. The difference between the extract, smoke, and smoke + extract groups was significant, but only the smoke group was significantly different from the control group (p˂0.05) (Figure 5 B).

## Discussion

Traditional herbal medicines are used as a treatment to improve sperm and semen parameters and increase the fertility of subfertile men. One of the *C. intybus *applications in traditional Iranian medicine is fertility enhancement (Dorostghoal et al., 2020 ▶). This study was designed to investigate the effect of hydroalcoholic extract of *C. intybus* leaf on germ cells, apoptosis, and oxidative stress after exposure of male Wistar rats to CS.

Based on our results, CS significantly reduced the number of Sertoli cells, spermatogonia, spermatocytes, and spermatids in seminiferous tubules of adult male rats compared to the control group. According to the expression of caspase-3 in testicular tissue, we observed that the number of apoptotic cells in rats exposed to CS increased significantly. On the other hand, we evaluated the levels of MDA and GSH in testicular tissue to investigate the oxidative stress caused by CS. MDA levels as an indicator of lipid peroxidation in testicular tissue of rats exposed to CS increased significantly. GSH is an antioxidant and its changes in the groups were different from changes in MDA levels so CS reduced GSH levels in testicular tissue. The expression of *Ahr* and *Cyp1a1* genes was studied in order to understand the mechanisms involved in damage caused by CS in testicular tissue. Exposure to CS significantly increased the expression of *Ahr* and *Cyp1a1* genes compared to other groups.

The effect of smoking on the reduction of mature sperm cell population can be direct (reduction of spermatogenic cells, death or destruction of growing cells) or indirect (developmental abnormalities due to apoptosis, tissue ischemia, oxidative stress, or other cases). CS causes a decrease in spermatogenic cells, irregular arrangement of cells in the seminiferous tubules, and a decrease of spermatozoa in the epididymal lumen and seminiferous tubules of mice (Omotoso et al., 2017 ▶). Esakky et al. (2012) ▶ observed disruption of the seminiferous tubules following *in vivo* exposure to cigarette smoke condensate (CSC), growth arrest, and death of spermatocytes after *in vivo* and *in vitro* exposure to CSC. They also found that CSC results in ROS accumulation, DNA damage, cell cycle arrest, and increased the expression of antioxidant enzymes in spermatocytes, and suggested the *Ahr-Nrf2* pathway activity for mentioned effects. Ahr activation by polycyclic aromatic hydrocarbons (PAHs) leads to cell cycle arrest, spermatogenic arrest, meiosis suppression, and spermatocyte death (Esakky and Moley, 2016b ▶). In this study, probably one of the reasons for stopping spermatogenesis and meiosis, reducing the number of different cells, and apoptosis in the seminiferous tubules of rats exposed to CS is upregulation of the *Ahr *expression by PAHs. These findings differ from the results of some other studies (Hansen et al., 2014 ▶; Huang et al., 2016 ▶).

Hansen et al. (2014) ▶ observed significant degeneration of seminiferous epithelium, compromised function of Sertoli cell, and germ cell defects (decreased spermatogonial proliferation and defects in mature germ cell formation) in *Ahr*^−/−^mice. AHR is found in the Sertoli cell nucleus, acrosome, and sperm flagellum. It modulates interactions between Sertoli and germ cells and plays a role in the development of fully functional mature sperm. Huang et al. (2016) ▶ stated that knockout of *Ahr* in mice causes testicular degeneration, germ cell apoptosis, reduced primary sperm cells, and reduced fertility. Reports of AHR’s role in apoptosis are conflicting. Some believe that it reduces apoptosis and others believe that it increases apoptosis (Esakky et al., 2015a ▶).

Esakky and Moley (2016a) ▶ found that PAHs are toxic to the testes. These compounds block spermatogenesis, increase apoptosis in the seminiferous tubules, and reduce testicular weight. AHR mediates the effect of PAHs on germ cells, thus it can serve as a therapeutic target and its inhibition is anti-apoptotic. AHR deficiency results in reduced male fertility, germ cell destruction, and defective function of Sertoli cells. ROS are involved in sperm maturation, but mature male gametes are very sensitive to oxidative stress because the expression of antioxidant enzymes in these gametes is low. AHR induces the expression of *Cyp1a1* and *Cyp1a2* genes in order to detoxify exogenous and endogenous ligands (Barouki et al., 2007 ▶). The high vulnerability of sperm to PAHs may be due to the presence of AHR (Esakky and Moley, 2016a ▶). Even at low CSC concentrations, the upregulation of *Cyp1a1* occurs AHR-dependently. CYP1A1 detoxifies PAHs but may cause germ cell death and apoptosis due to the production of benzopyrene diol epoxide (BPDE). CSC can cause germ cell death by causing AHR-mediated oxidative stress, and over-induction of AHR by CSC is an undesirable event, thus such pathways must be blocked to prevent the accumulation of ROS and other unwanted metabolites (Esakky and Moley, 2016a ▶).

As mentioned, AHR can be a therapeutic target to prevent the adverse effects of CS on germ cells. In the present study, we evaluated the effects of *C. intybus *leaf extract on CS damage in the testes of Wistar rats. Treatment with *C. intybus *leaf extract compensated for the reduction of various cells in the seminiferous tubules and the number of Sertoli cells, spermatogonia, spermatocytes, and spermatids was even higher in the extract group compared to the control group, although this increase was not significant. We also observed that treatment with the extract reduced apoptosis, and even the rate of apoptosis in the group treated with *C. intybus *leaf extract alone was lower than the control group. Hydroalcoholic extract of *C. intybus *leaf alone and in combination with CS caused a significant reduction in MDA levels so that they were not considerably different from the control group. A notable increase in GSH levels was observed in the groups treated with the *C. intybus *extract compared to the smoke group. Simultaneous treatment with *C. intybus *leaf extract caused the expression of *Ahr* and *Cyp1a1* to be lower than the control group.

Mathur et al. (2015) ▶ used *C. intybus* leaf extract to prevent N-nitrosodiethylamine (NDEA)-induced hepatotoxicity. NDEA is one of the carcinogens of tobacco smoke. The results of the treatment showed that levels of antioxidant enzymes such as glutathione reductase (GR) and glutathione peroxidase (GPx) were markedly increased. It can be said that *C. intybus* leaf extract plays a protective role against NDEA by reducing oxidative stress. Hassan and Yousef (2010) ▶ have also confirmed the role of *C. intybus*as as a natural substance in improving oxidative stress and liver damage caused by nitrosamine. This effect may be due to the upregulation of endogenous antioxidant defense and the elimination of free radicals.

Esakky et al. (2015a) ▶ remarkably attenuated the proapoptotic effect of CSC in GC-2spd(ts) cells by AHR inhibitor CH223191 (an AHR antagonist). While suppression of *Ahr* alone significantly increased spermatocyte apoptosis. In the study by Esakky et al. (2015a) ▶, the expression percentage of activated caspases-3 and 7 in SCS-exposed spermatocytes increased to about 68%. AHR inhibition by using CH223191 resulted in a significant reduction of caspase-3/7 expression in spermatocytes. Inhibitor blocks activation of AHR, and subsequently blunts caspase activation due to CSC.

In the present study, downregulation of *Ahr *and *Cyp1a1*, a decrease of oxidative stress, and reduction of apoptosis by *C. intybus* leaf extract occurred. The *C. intybus *extract probably affected *Cyp1a1* expression by downregulation of *Ahr*, and downregulation of these two genes led to a lack of accumulation of undesirable metabolites, including ROS. On the other hand, decreasing MDA levels and increasing GSH levels showed a decrease in free radicals and an improvement in antioxidant status, respectively. By reducing oxidative stress and apoptosis, the structure of seminiferous tubules improved and cell death decreased.

The main therapeutic and pharmacological properties of *C. intybus* are related to its antioxidant activity due to the presence of phenolic compounds such as some flavonoids (Bahmani et al., 2015 ▶). Our results confirmed the protective role of *C. intybus *leaf extract against the adverse effects of CS on the testis. Exposure to CS can cause oxidative stress damage and increase cell apoptosis, so the use of antioxidants may be a useful defense against the toxicity of CS. The effects of *C. intybus* extract may be related to its antioxidant properties and downregulation of *Ahr*, however, signaling pathways affected by *Ahr* should be considered to comment on this with more confidence. Our data confirm the traditional use of *C. intybus* for treatment of male reproductive problems. Further studies are needed to elucidate the exact mechanism of action of *C. intybus* extract and to distinguish between the roles of different compounds in the extract and their toxicity or therapeutic effects. 

## Conflicts of interest

The authors have declared that there is no conflict of interest.
